# Identifying immune checkpoint-related lncRNA biomarkers for immunotherapy response and prognosis in cancers

**DOI:** 10.1038/s41597-023-02550-z

**Published:** 2023-09-28

**Authors:** Yue Gao, Xinyue Wang, Longlong Dong, Changfan Qu, Qianyi Lu, Peng Wang, Mengyu Xin, Wen Zheng, Chenyu Liu, Shangwei Ning

**Affiliations:** College of Bioinformatics Science and Technology, Harbin Medical University, Harbin, 150081 China

**Keywords:** Immunotherapy, Long non-coding RNAs

## Abstract

Long non-coding RNAs (lncRNAs) could modulate expression of immune checkpoints (ICPs) in tumor-immune. However, precise functions in immunity and potential for predicting ICP inhibitors (ICI) response have been described for only a few lncRNAs. Here, a multiple-step pipeline was developed to identify cancer- and immune-context ICP and lncRNA cooperative regulation pairs (ICPaLncCRPs) across cancers. Immune-related ICPs and lncRNAs were extracted follow immune cell lines and immunologic constant of rejection groups. ICPaLncCRP networks were constructed, which likely to modulate tumor-immune by specific patterns. Common and specific hub ICPaLncs such as MIR155HG, TRG-AS1 and PCED1B-AS1 maybe play central roles in prognosis and circulating. Moreover, these hub ICPaLncs were significantly correlated with immune cell infiltration based on bulk and single-cell RNA sequencing data. Some ICPaLncCRPs such as IDO1-MIR155HG could predict three- and five-year prognosis of melanoma in two independent datasets. We also validated that some ICPaLncCRPs could effectively predict ICI-response follow six independent datasets. Collectively, this study will enhance our understanding of lncRNA functions and accelerate discovery of lncRNA-based biomarkers in ICI treatment.

## Introduction

Cancer treatment has radically changed over time, evolving from a one-size-fits-all approach to a more tailored, personalized approach. In recent years, immunotherapy, especially the application of immune checkpoint inhibitors (ICIs) has revolutionized the treatment for a range of cancer types^1,2^. ICIs are monoclonal antibodies developed for corresponding immune checkpoints (ICPs). Their main role is to block the interaction between tumor cells expressing ICPs and immune cells, thus blocking the inhibition of tumor cells on immune cells^3^. Although immunotherapy being heralded as a turning point in cancer care, low response rate, immune related adverse events and different degrees of drug resistance greatly limit the clinical application of ICI^4,5^. Thus, it is urgent to identify effective and accurate biomarkers for predicting ICI response.

Long noncoding RNAs (lncRNAs) is a class of non-coding RNAs which their transcripts are longer than 200 nucleotides with no protein-coding capacity^6,7^. They are widely studied and well-known because of their important regulatory roles and extensive functional diversity in both cellular and developmental processes through influencing gene expression at epigenetic, transcriptional and posttranscriptional levels^8,9^. Dysregulation of lncRNAs in cancer can be used as biomarkers for diagnosis and potential targets for cancer therapeutics^10–12^. Furthermore, the latest and growing research had revealed that the relevance of lncRNAs in the development and function of the immune system^13,14^. More and more evidence reported that lncRNAs had colossal potential to evaluate ICI response and predict clinical outcomes^15,16^. Based on high-throughput sequencing expression profile and bioinformatics technology, lncRNAs could become powerful ICI response and prognostic biomarkers in cancers.

ICPs exist in various immune cells such as natural immune cells including monocyte cell, NK cell, neutrophil cell and adaptive immune cells including cytotoxic T cell and T helper cell. Further targets are constantly being added and it is becoming increasingly clear that their expression is not only relevant on T cells^17^. Recent studies had rapidly identified a number of ICPs on diverse immune cells. However, most of these ICPs had not been deeply studied and applied to clinical treatment. LncRNAs, an important class of gene regulation regulators, may contribute to the innate and adaptive immune activities^18^. Xu *et al*. reported that HCP5 and MIAT promoted tumor growth and upregulated the expression of PD-L1/CD274 via a competing endogenous RNA (ceRNA) mechanism of sponging miR-150-5p. However, widely scale of associations between ICPs and lncRNAs is unclear, and the landscape of ICPaLncs in human cancer is not known.

To systematically explore the crosstalk among lncRNAs, ICPs and immunity, cancer- and immune-context ICP and lncRNA cooperative regulation pairs (ICPaLncCRPs) were identified based on an integrated pipeline (Figure S1) including expression pattern analysis across immune cell, immunologic constant of rejection (ICR) and co-expression. We found common and specific ICP-associated lncRNA (ICPaLnc) hubs were involved in multiple levels of cancer processes such as survival and circulating. ICPaLncCRPs were correlated with immune cell infiltration, especially T and B cells based on bulk and single-cell RNA sequencing. Specially, some ICPaLncCRPs could become as potential biomarkers for predicting prognosis and ICI response in skin cutaneous melanoma (SKCM) based on multiple independent datasets. In summary, our method provides a system pipeline to unveil lncRNA biomarkers for ICI-treated patients, helping previously identified biomarkers to improve the prediction of the ICI response.

## Results

### Immune-related ICPs and lncRNAs are identified and characterized across immune cell types

**Fig. 1:**
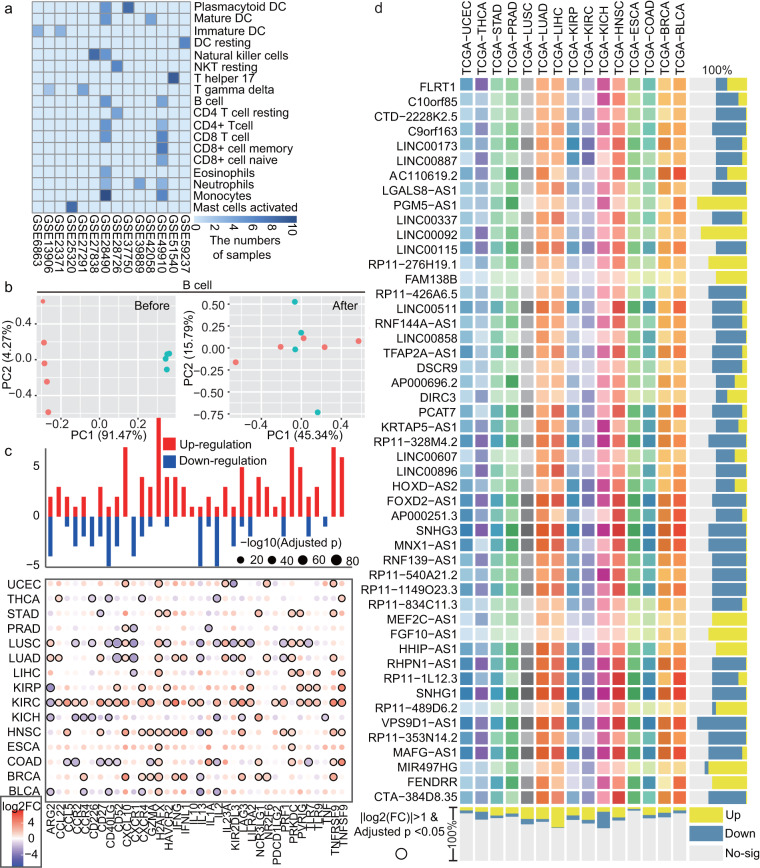
Some ICPs and lncRNAs are highly expressed in multiple immune cell types. (**a**) The heatmap shows numbers of samples for different immune cell types after removing batch effect in diverse datasets. (**b**) The point plots show two datasets (pink and blue-green) before and after removing batch effect in B cell. (**c**) Top bar chart shows up- (red) and down-regulated (blue) highly expressed ICPs between cancer and normal tissues in TCGA. The bubble plot shows fold change and P values of differential expressed ICPs across cancer types. (**d**) The heatmap shows dysregulation of highly expressed lncRNAs across cancer types. Right bar chart shows up- (yellow), down- (blue) regulation and non-significance (Grey).

In order to identify highly expressed ICPs and lncRNAs in immune cells, 18 immune cell types were integrated and removed batch. The numbers of samples for different immune cell types in diverse datasets showed distinctions (Fig. 1a). The immune cell expression profile data showed more decentralized and average distribution after removing batch (Fig. 1b, Figure S2). Many ICPs and lncRNAs were highly expressed in multiple kinds of immune cells in immune cell lines data (Fig. 1c). Up- and down-regulated patterns between tumor and normal tissues in TCGA data were diverse in cancers. For example, 27 ICPs and 650 lncRNAs were highly expressed in 18 immune cell types (Figure S3a). Two kinds of methods including ESTIMATE and ssGSEA were used to extract TCGA samples with high immune infiltration for follow analysis (Fig. 1d, Figure S3b). Thus, highly expressed ICPs, lncRNAs in TCGA samples with high infiltration across immune cell types were extracted.

**Fig. 2:**
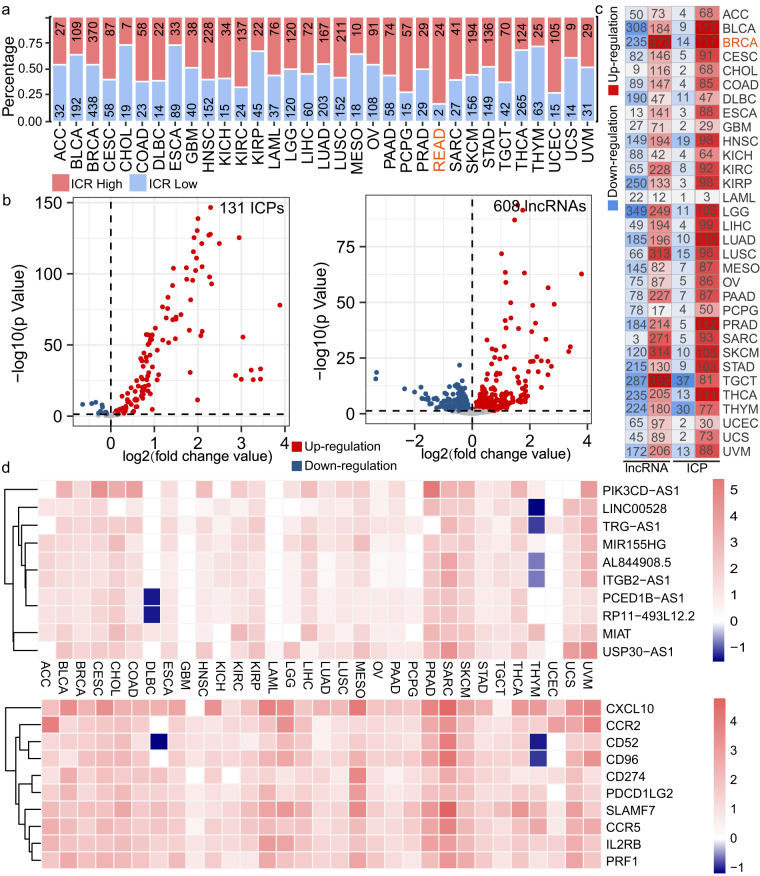
Immune-related ICPs and lncRNAs are identified and characterized across immune cell types. (**a**) The bar plot shows numbers of samples in high (red) and low (blue) ICR groups across cancer types. (**b**) The two volcano plots show differentially expression of immune-related ICPs and lncRNAs between high and low ICR groups in BRCA. (**c**) The heatmap shows up- and down-regulated immune-related ICPs and lncRNAs between cancer and normal tissues in TCGA. (**d**) The heatmaps show top 10 differential expressed immune-related ICPs and lncRNAs across cancer types.

In order to identify immune-related ICPs and lncRNAs, highly immune infiltrated samples were divided high and low ICR groups (Figure S4). The numbers of high and low ICR samples were diverse in different cancer types (Fig. 2a). Rectal cancer (READ) had most highest proportion of samples with high ICR. Differential expressed immune-related ICPs and lncRNAs were identified between high and low ICR groups (Fig. 2b). There were most up-regulated immune-related ICPs and lncRNAs in breast cancer (BRCA). The numbers of up-regulated ICPs and lncRNAs were far higher than down-regulated ICPs and lncRNAs (Fig. 2c). These results indicted that immune-related ICPs and lncRNAs maybe showed immune activation status in BRCA. The most differential expressed immune-related ICPs and lncRNAs were up-regulated in other cancer types except Lymphoid Neoplasm Diffuse Large B-cell Lymphoma (DLBC) and thymoma (THYM) (Fig. 2d). Collectively, these results imply that the immune-related ICPs and lncRNAs might play critical roles in the tumor-immune microenvironment.

### ICPaLncCRPs show specific regulation relationships in cancers

**Fig. 3:**
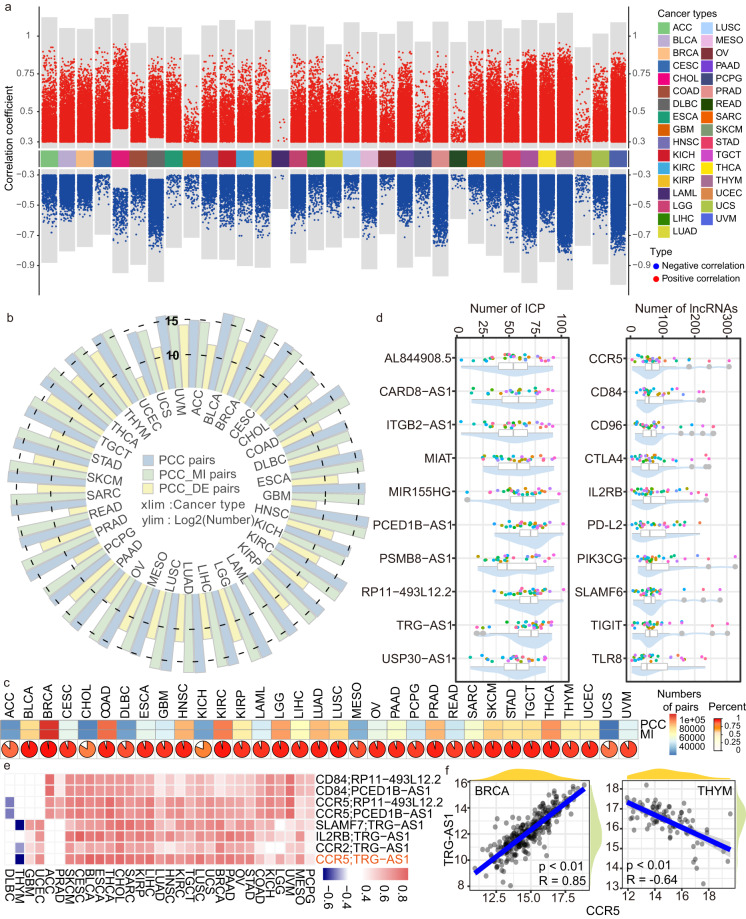
ICPaLncCRPs show specific regulation relationships. (**a**) The Manhattan plot shows positive (red) and negative (blue) correlated ICPaLncCRPs across cancer types. (**b**) The circular bar plot shows numbers of ICPaLncCRPs extracted by PCC (blue), intersection of PCC and MI (green) and differential expressed pairs (yellow) across cancer types. (**c**) The heatmap shows numbers of ICPaLncCRPs extracted based on PCC and MI. The pie charts show intersections between PCC and MI. (**d**) The violin plots show the numbers of interacted ICPs and lncRNAs for some lncRNAs and ICPs across cancer types. (**e**) The heatmap shows PCC values of ICPaLncCRPs which were present in multiple cancer types. (**f**) The point plots show correlations between CCR5 and TRG-AS1 in BRCA and THYM.

ICPaLncCRPs were extracted and ICPaLncCRPs co-expressed networks were constructed such as BRCA and SKCM (Fig. 3a, Figure S5a). Similar to other biological networks, ICPaLncCRP co-expressed network follow the power-law distribution, and have characteristics of scale-free in BRCA (R^2^ = 0.858) and SKCM (R^2^ = 0.741, Figure S5b). The related patterns including positive and negative correlations were diverse in different cancer types. Two co-expression methods including PCCs and MI methods showed higher interactions (Fig. 3b). The coincidence rate was above 75% in almost all cancers (Fig. 3c). The interaction patterns between ICP and lncRNA show complex and close relationships. Key differential immune-related ICPs and lncRNAs could participate in many co-expressed pairs in diverse cancer types (Fig. 3d). For example, ICP gene CD96 had 259, 257, 225, 191 and 157 cooperative lncRNAs in THYM, UVM, Testicular Cancer (TGCT), Pancreatic Cancer (PAAD) and Lower Grade Glioma (LGG). We also found lncRNA MIAT was a key lncRNA which could participate in many ICPaLncCRPs in diverse cancer types including THYM, SKCM, Thyroid Cancer (THCA), UVM and Bladder cancer (BLCA). Previous study had demonstrated that the combination of MIAT knockdown and PD-L1 antibody administration showed a synergistic inhibitory effect on tumor growth^19^. Some ICPaLncCRPs were presented in many kinds of cancer types (Fig. 3e). Similar to differential expressed immune-related ICPs and lncRNAs, some ICPaLncCRPs also showed specific negative correlations in DLBC and THYM. For example, ICP CCR5 and lncRNA TRG-AS1 were positive correlated in BRCA (PCC = 0.85, P value < 0.01) but negative correlated in THYM (PCC = −0.64, P value < 0.01) (Fig. 3f). These results indicated that ICPaLncCRPs were present in most cancers and show specific regulation patterns.

### Common and specific ICPaLnc hubs are involved in multiple levels of cancer processes

**Fig. 4:**
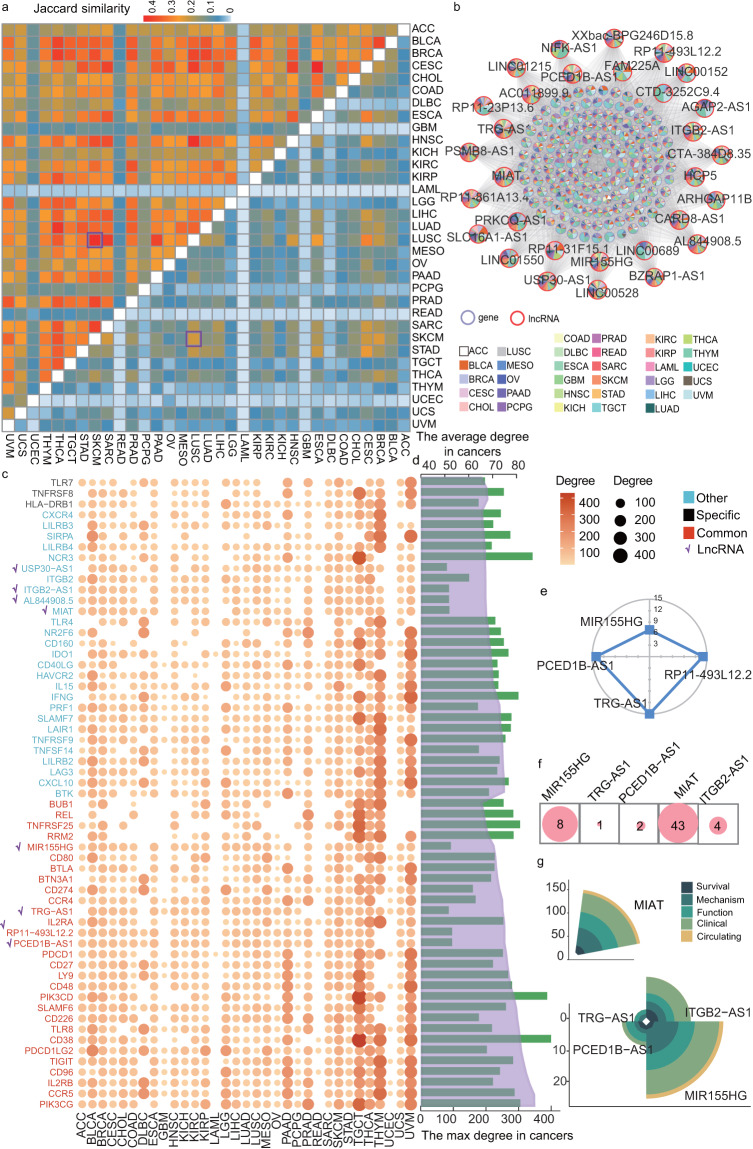
Common and specific ICPaLnc hubs are involved in multiple levels of cancer processes. (**a**) The upper triangular matrix shows the proportion of ICPs and ICPaLncs shared by any two cancer types. The lower triangular matrix shows the proportion of shared ICPaLncCRPs. (**b**) The hub ICPaLnc network in pan-cancer. Purple and red nodes on the outer circle indicate ICPs and ICPaLncs, a color pie chart shows cancers in which the ICP or ICPaLnc occur and size of the node shows the degree of nodes. An edge indicates a cooperative regulation between ICPs and ICPaLncs. (**c**) The classification of hub ICPs and ICPaLncs in pan-cancer. The bubble diagram indicates the degree of each hub ICP and ICPaLnc across cancer types. (**d**) The bar plot in green color shows the max degree of each hub ICP and ICPaLnc in the pan-cancer. The line plot in purple shows the average degree of each hub ICP and ICPaLnc in the pan-cancer. (**e**) The radar chart shows numbers of cancer types which the lncRNAs are present in. (**f**) The circles represent numbers of verified literature related with cancers for hub ICPaLncs. (**g**) The rose diagram represents numbers of verified literature related with cancer process for hub ICPaLncs.

To further gain insight into the roles of ICPaLncCRPs in immunity and cancer development, we extracted all the ICPaLncCRPs in each cancer types. A similarity matrix was constructed based on shared ICPs, ICPaLncs and ICPaLncCRPs between any two cancer types (Fig. 4a). Some cancers shared more ICPaLncCRPs. A higher number of ICPaLncCRPs were identified in the cancer types where ICI drugs were applicable in clinical. For example, SKCM and LUSC shared 2389 common ICPaLncCRPs. These 2389 IC-lncRNAs account for ~52.85% of all ICPaLncCRPs in LUSC. A few nodes with a large number of neighbors as hubs hold the nodes together in each network. Almost all biological networks presented the general feature hubs. To investigate the crucial nodes in the ICPaLncCRPs network of pan-cancer, top 5% of the nodes with the highest degree were considered as hub ICPs or lncRNAs. The hub ICPs and lncRNAs networks in pan-cancer were constructed (Fig. 4b, Figure S6). Hub lncRNAs in pan-cancer network were present in higher number of cancer types. Then, we investigated the roles of these hub ICPs and lncRNAs across cancer types and grouped these hubs into three categories: common hubs, specific hubs, and other hubs. The common hubs signify the central roles of ICPaLncCRPs in more than five cancer; the specific hubs identify ICPaLncCRPs with specific central roles in a given cancer. Four common and four specific hub lncRNAs were identified across cancer types (Fig. 4c). The max and average degree of these common hubs distributed higher level (Fig. 4d). The four common hub lncRNAs MIR155HG, PCED1B-AS1, RP11-493L12.2 and TRG-AS1 were present in 7, 14, 14 and 15 cancer types, indicated they played crucial roles in immunity and cancer process (Fig. 4e). Some hub and specific lncRNAs had been verified were associated with cancers obtained from Lnc2Cancer 3.0 (Fig. 4f). Specially, MIAT and MIR155HG could be detected in blood of cancer patients (Fig. 4g). To together, some of our identified ICPaLncCRPs were associated with immunity and cancer development and ICPaLncs seem to become effective biomarkers for cancers.

### ICPaLncCRPs are correlated with immune cell infiltration based on bulk and single-cell RNA sequencing

**Fig. 5:**
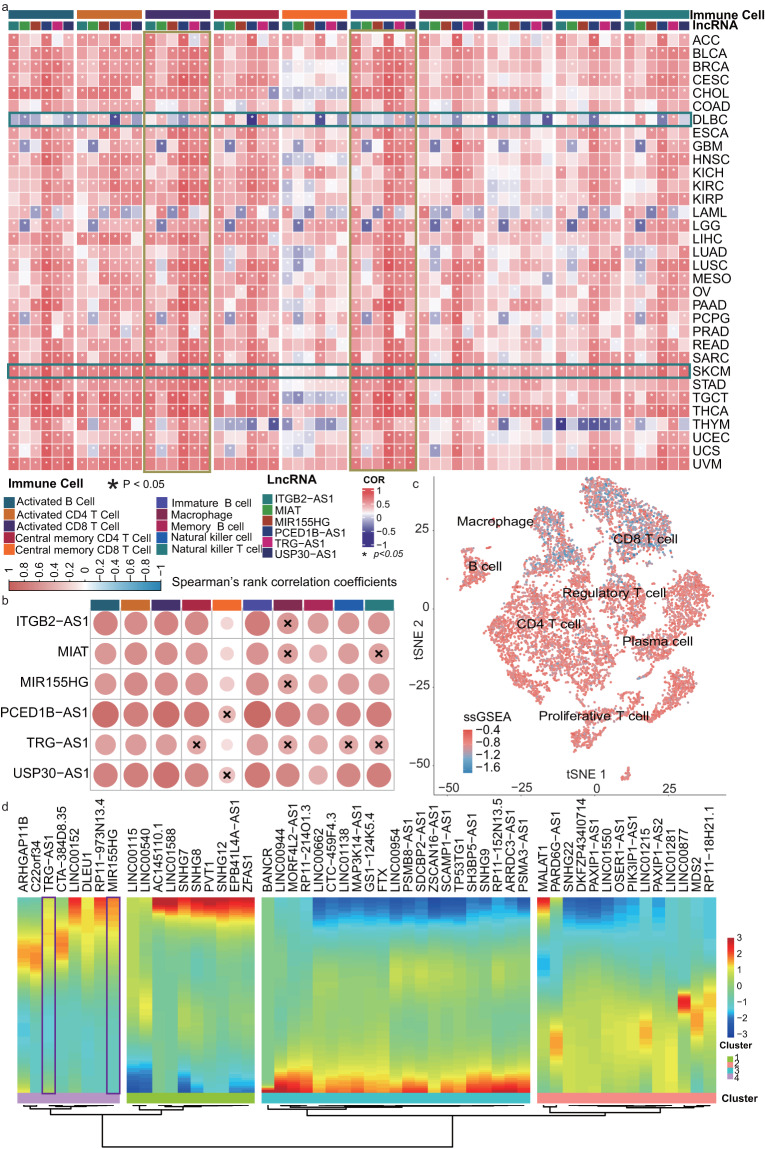
ICPaLncCRPs are correlated with immune cell infiltration based on bulk and single cell sequencing. (**a**) The heatmap shows Spearman’s rank correlation coefficients between expression of hub lncRNAs and immune cell types across cancer types, * indicate p < 0.05. (**b**) The Spearman’s rank correlation coefficients between some hub lncRNAs and immune cell types in SKCM. (**c**) tSNE plot of immune cells, color-coded by ssGSEA scores for ICP-related lncRNAs in SKCM. (**d**) The heatmap shows pseudotime of ICPaLncs in SKCM.

The complexity and diversity of the immune context of the tumor microenvironment is broadly populated with immune cells^20^. Therefore, we reasoned that if these ICPaLncCRPs participate in tumor immune microenvironment and ICI treatment regulation, then they would be more likely to have higher expression in immune cells and to be related with immune cell infiltration in tumors. We found that hub ICPaLncs were correlated with many kinds of immune cell types, especially T cells and B cells (Fig. 5a). Hub ICPaLncs were correlated with immune cells infiltration in most cancers. However, DLBC was still a special cancer similar to previous analysis and showed lower immune infiltration. Compared to other cancers such as BRCA, hub ICPaLncs were more correlated with immune cells infiltration in SKCM (Fig. 5b, Figure S7a). Similar results were also found in single cell dataset of SKCM. The resulting 13,659 cells were clustered into eleven major cell types and further into seven subtypes (Figure S7b). We found ssGSEA scores of ICPaLncs were different in CD8+ T cells (Fig. 5c). These ICPaLncs were dynamically changed in diverse pseudotime points (Fig. 5d). All cells formed a branched structure, with four transcriptional states based on expression of ICPaLncs (Figure S7c,d). In summary, these results suggested that ICPaLncs exhibited higher expression in immune cells and were associated with immune cell infiltration, further validating the roles of the ICPaLncCRPs in tumor-immune microenvironment.

### ICPaLncCRPs have an effect on the prognosis of cancer patients

**Fig. 6:**
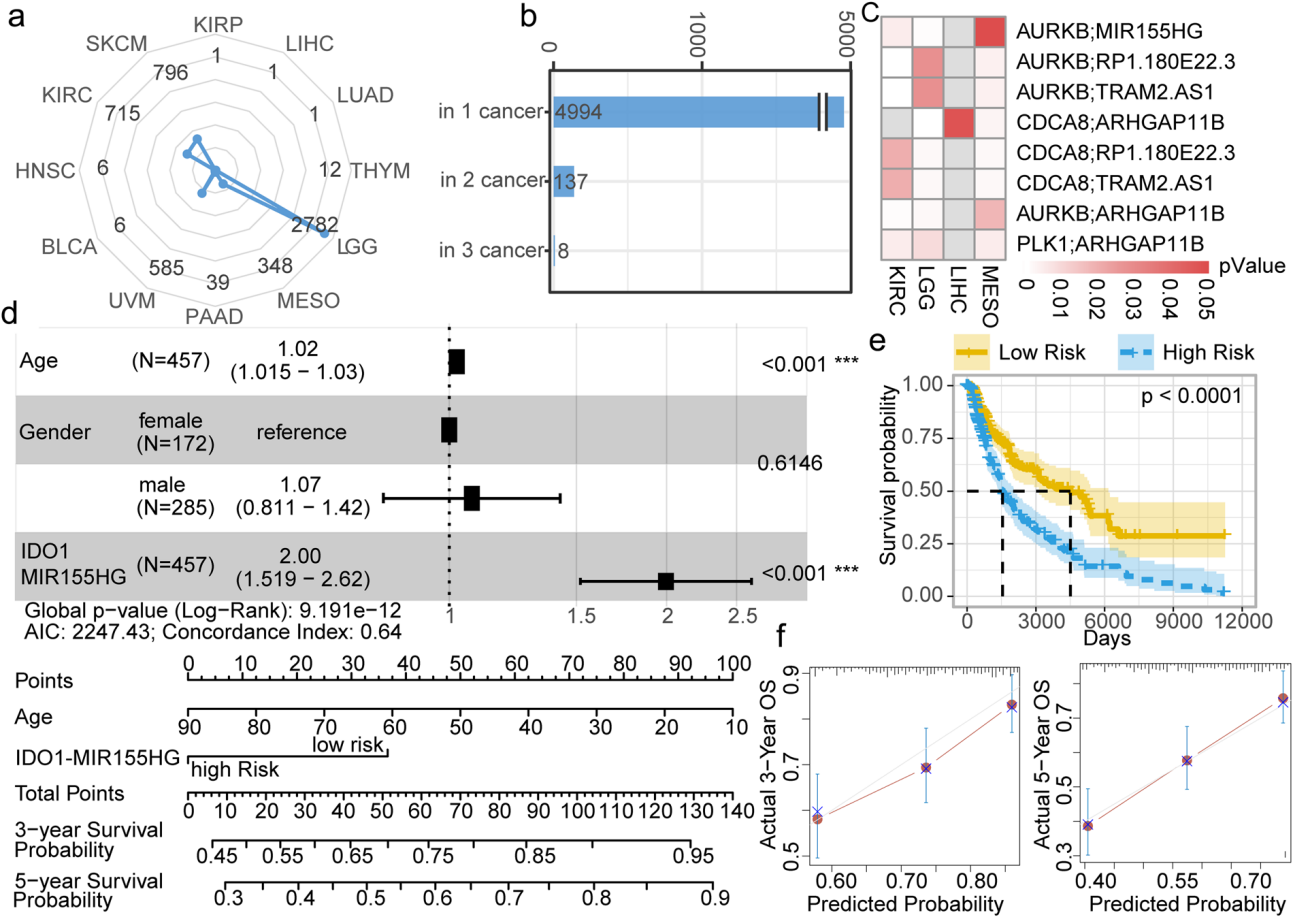
ICPaLncCRPs have an effect on the prognosis of cancer patients. (**a**) The radar chart shows numbers of survival-associated ICPaLncCRPs across cancer types. (**b**) The bar plot shows numbers of survival-associated ICPaLncCRPs related to one, two and three kinds of cancer types. (**c**) The heatmap shows P values of survival-associated ICPaLncCRPs in three cancer types. (**d**) The forest plot shows that the signature (ICPaLncCRPs: IDO1-MIR155HG) is independent from other risk factors for prognostic prediction. Nomogram to estimate the prognostic risk of IDO1 and MIR155HG pair. Each variable axis, containing age and corresponded to the characteristic attribute score of single sample. The likelihood of 3- and 5 years OS is determined on the survival axis. (**e**) Kaplan-Meier survival analysis of the OS for patients with high (blue) and low (yellow) risk scores. (**f**) The calibration curves yield an accurate predictive capability that was extremely close to actual survival (3- and 5 years OS) are presented by the calibration plots. X-axis represents the predicted value of survival probability and y-axis represents actual survival possibility.

Numerous studies elucidate how various components of the immune system control or contribute to cancer progression, thus revealing their prognostic value^21^. We next investigated whether these ICPaLncCRPs were associated with the survival of cancer patients. A comprehensive pipeline based on risk score and permutation was performed to extract survival-related ICPaLncCRPs. Large different amounts of the survival-related ICPaLncCRPs were identified in diverse cancer types (Fig. 6a). More survival-related ICPaLncCRPs were identified in LGG, SKCM, Kidney renal clear cell carcinoma (KIRC) and Uveal Melanoma (UVM) which had been treated by ICI in clinical. We could infer that higher number of ICPaLncCRPs were associated with prognosis in the cancer types where ICI drugs were applicable in clinical^22–25^. Most ICPaLncCRPs showed cancer specific and were associated with prognosis in only one cancer type (Fig. 6b). However, there were eight ICPaLncCRPs could play as prognostic biomarkers in three kinds of cancer types (Fig. 6c). CDCA8, AURKB and PLK1 were also three hub ICPs in multiple kinds of cancers. LncRNA MIR155HG was also a key hub lncRNA in all kinds of cancers. In our analysis, ICPaLncCRP AURKB-MIR155HG was associated with survival in KIRC, LGG and MESO. Some previous study also reported that MIR155HG is a prognostic biomarker and associated with immune infiltration and immune checkpoint molecules expression in multiple cancers^26^. In SKCM, ICPaLncCRP IDO1-MIR155HG was demonstrated an independent prognostic factor by forest plot for overall survival (OS), suggesting it could become as a key prognostic biomarker (Fig. 6d). Based on the independent predictors obtained from the multivariate analysis, a nomogram was established to predict 3- and 5-year OS. ICPaLncCRP IDO1-MIR155HG exerted the largest effect on the OS, with a maximal score of 140 points. Lower risk score of IDO1-MIR155HG showed better prognosis in SKCM (Fig. 6e). IDO1-MIR155HG was also associated with survival in an independent dataset (Figure S8). The calibration curve showed that risk score of IDO1-MIR155HG had a satisfactory fit between the predictive and actual observations. Thus, the risk score of IDO1-MIR155HG could effectively predict three- and five-year prognosis of SKCM patients (Fig. 6f). Collectively, these results suggest that the cooperative pattern based on the expression of ICPaLncCRPs could effectively identify prognostic biomarkers for cancers.

### ICPaLncCRPs display different immune context and could predict ICI response in SKCM

**Fig. 7:**
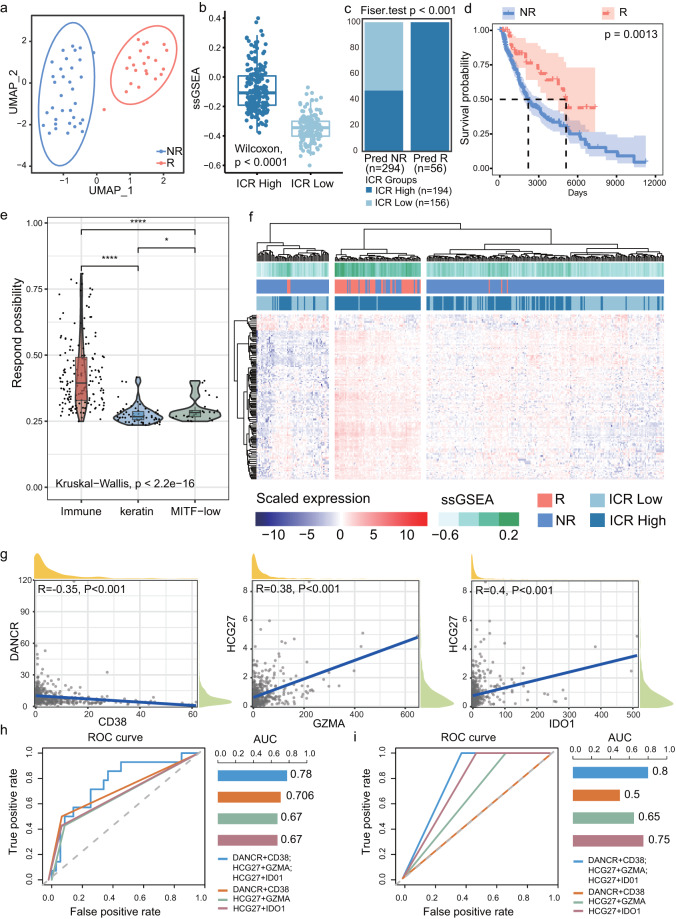
ICPaLncCRPs display different immune context and could predict ICI response in SKCM. (**a**) The UMAP clustering diagram shows responders (R, red) and non-responders (NR, blue). (**b**) The box plot shows ssGSEA scores of ICPaLncCRPs in high and low ICR groups. (**c**) ICI response prediction using the expression levels of ICPaLncCRPs. Predicted responders (Pred R) and non-responders (Pred NR) are plotted against high ICR (dark blue) and low ICR group (light blue). The two-sided Fisher’s exact test is used to compute statistical significance. (**d**) Kaplan-Meier survival analysis of the OS for responders (red) and non-responders (blue). (**e**) The violin plot shows respond possibility in ‘immune’ (red), ‘keratin’ (blue) and ‘MITF-low’ (green) clusters of SKCM. (**f**) The heatmap shows expression of ICPaLncCRPs in SKCM.

In order to evaluate predictive performances of ICPaLncCRPs to predict the ICI response, multiple datasets about SKCM were used for analysis. The ICI responders and non-responders could be well distinguished after UMAP dimension reduction and clustering based on the expression of ICPaLncCRPs in SKCM dataset GSE35640^27,28^ (Fig. 7a). SsGSEA scores of ICPaLncCRPs were significantly higher in high ICR group than low ICR group (P < 0.0001, Fig. 7b). It indicated that ICPaLncCRPs could report the immune context of patient. We conducted an SVM model to measure the performance using ICPaLncCRPs biomarkers. The SVM model was trained by SKCM dataset GSE35640 and tested by TCGA SKCM dataset. We found all the predicted responders were in high ICR group (P < 0.001, Fig. 7c). Furthermore, a prolonged overall survival was consistently observed for patients predicted as ICI responders using ICPaLncCRPs based on SVM model in TCGA SKCM dataset (P = 0.0013, Fig. 7d). SKCM patients in TCGA could be clustered to three subtypes including ‘immune’, ‘Keratin’ and ‘MITF-low’ clusters^29^. The immune cluster had significant outcome correlation with genomic classification, and was associated with lymphocyte infiltrate on pathology review and high LCK protein expression, a T cell marker. The ‘immune’ cluster was also associated with improved patient survival. In our analysis, patients with the ‘immune’ cluster in the SKCM TCGA dataset were likely to be predicted ICI responders based on ICPaLncCRPs, suggesting that predicted ICI responders have high immune infiltration levels (P < 0.0001, Fig. 7e). The resonders and non-responders, high and low ICR groups could be clustered together based on ICPaLncCRPs (Fig. 7f). There were more reponders, high ICR patients and higher ssGSEA scores in cluster 2, suggesting these patients could become better candidates for ICI treatment. We also extracted three ICPaLncCRPs including DANCR-CD38, HCG27-IDO1 and HCG27-GZMA to predict ICI response based on LASSO regression. The three ICPaLncCRPs showed strong correlations (Fig. 7g). The predicted ability of three integrated ICPaLncCRPs (AUC = 0.775) was higher than single ICPaLncCRP in GSE35640 (Fig. 7h). Similar results were also got in five another independent datasets (Fig. 7i). Collectively, the integrated pipeline effectively select ICI response-associated ICPaLncCRPs biomarkers that can make robust predictions for precision oncology in ICI treatment.

## Discussion

Dysfunction of the ICPs in tumor cells enables them to evade recognition and killing by immune cells, thus promoting tumor growth and metastasis^30^. Accumulating evidence suggests that lncRNAs could be involved in regulating ICPs. However, systematic analysis of ICP-related lncRNA is lacking especially in cancers, especially their potential in predicting prognosis and ICI response. Herein, an integrated computational pipeline was developed to identify ICP-related lncRNAs across cancers (Figure S1). Our results demonstrated a complex relationship between ICP, lncRNA and immunity, help to evaluate ICI response for cancer patients.

Immunity is a common hallmark for cancers. In our analysis, immune activation were present in most cancer types. However, DLBC and THYM were two special cancer types on multiple levels. For example, the most differential expressed immune-related ICPs and lncRNAs were down-regulated in DLBC and THYM. The great majority of correlations between ICPs and lncRNAs in ICPaLncCRPs were negative in DLBC and THYM. Hub lncRNAs were lightly correlated with immune cells infiltration in DLBC. DLBC and THYM are two cancers highly related to immunity. We also found 12 ICPaLncCRPs were related to survival in THYM. The results indicated that pattern of lncRNAs in ICP regulation were more complex and specific in DLBC and THYM.

Unprecedented breakthroughs have been achieved in cancer treatment with the emergence of immune checkpoint blockade immunotherapy^30^. Durable benefits have been produced by inhibiting PD-1, PD-L1, CTLA-4. Further targets are constantly being added and it is becoming increasingly clear that their expression is not only relevant on T cells^17^. In our analysis, we try to cover the ICPs in the current clinical application and experimental stage. Despite all the achievements, only a limited number of patients benefited from ICI treatment. Ongoing studies have been devoted to explain the underlying mechanisms of adverse events and different response after ICI treatment. However, most current studies only focused on several essential ICP genes; the relationship between lncRNAs and ICI responses has not received much attention. We systematically analyzed ICP-related lncRNAs in pan-cancer, and found that the potential clinical application of ICPaLncCRPs is different among cancer types. SKCM is a highly aggressive form of skin cancer, where it is often difficult to treat with traditional therapies. Poor long-term prognosis and elusive biomarkers were key problems for exploring mechanism and treatment for SKCM. We found IDO1-MIR155HG pair could become effective biomarker for predicting three- and five-year prognosis of SKCM patients in two independent datasets. Furthermore, ICPaLncCRPs could evaluated the response of ICI in SKCM based on six independent datasets. SKCM patients with ‘Immune’ subtype were more likely to be responders of ICI. Our findings revealed the indispensable roles of ICP-related lncRNAs in cancer immunotherapy.

In our study, we only focus on whole regulatory relationships between lncRNAs and ICPs. However, reveal the causal inferences between ICPs and lncRNAs is very essential and key. We try to infer the causal inferences using Bayes network and Maximum likelihood estimation which had been used for some previous studies in SKCM^31,32^. The relationship of lncRNA regulating ICP were appears in nearly half of all the ICPaLncCRPs (Figure S9). More data, computational method, *in vivo* and *in vitro* experiments should be performed to explore the causal relationship between ICP and lncRNA.

In summary, we presented the ICP and lncRNA regulation landscape across major human cancers and showed the importance of immunity. Our study opens new avenues to investigate the functions and mechanisms of lncRNAs in immune regulation, especially ICP regulation in tumors. Follow-up investigation is warranted to deepen our understanding of lncRNAs cancer immune functions and their application in immunotherapy.

## Methods

### Collection of clinical and experimentally verified ICPs

In order to identify ICP genes, we searched PubMed using a list of keywords, such as ‘immune checkpoint’, ‘immunotherapy’ and ‘ICP’. Also, we collected ICPs by handbooks or instruction of website from multiple companies. Only clinical and experimentally verified ICPs were collected.

### Obtaining expression profiles of purified immune cell types

Expression profiles of diverse immune cell types were referenced from a previous study^16^. Microarray data (Affymetrix HG-U133_Plus 2.0 platform) of B cells and 18 other immune cell types were obtained from the GEO Gene Expression Omnibus (GEO, https://www.ncbi.nlm.nih.gov/geo) database under the accession numbers GSE42058^33,34^, GSE49910^35,36^, GSE51540^37,38^, GSE59237^39,40^, GSE6863^41,42^, GSE8059^43,44^, GSE13906^45,46^, GSE23371^47,48^, GSE25320^49,50^, GSE27291^51,52^, GSE27838^53,54^, GSE28490^55,56^, GSE28698^57,58^, GSE28726^59,60^, GSE37750^61,62^ and GSE39889^63,64^.

### Extraction of highly expressed ICPs and lncRNAs across immune cell types

Highly expressed ICPs and lncRNAs were extracted across 18 immune cell types based on immune cell lines (Figure S1a). We defined the lncRNAs and ICPs which were highly expressed in immune cell lines as immune highly expressed ICPs and lncRNAs. Quantile normalization, background correction, and log2 transformation of microarray data from the Affymetrix platform were performed by the Robust Multi-array Average (RMA) algorithm^65^ from the R package affy. We renamed the probes of microarray data from the Affymetrix platform to get lncRNA expression profiles. Probe sets with Ensembl gene IDs as ‘long non-coding RNA’ was extracted after matching the annotation file of GENCODE (https://www.gencodegenes.org/, release 39) with the NetAffx annotation(https://www.affymetrix.com/analysis/netaffx_analysis_center_retired.html, release 36). Then, all the lncRNAs and ICPs were respectively ranked based on expression in each immune cell. In order to consider particular immune-related lncRNAs, only the lncRNAs which were present in a specific or multiple kinds of immune cells were extracted. The lncRNAs which were highly expressed (top 50%) at one kind of immune cell (immune-specific lncRNA) or more than nine kinds of immune cells (immune-general lncRNA) were screened. The ICPs which were highly expressed (top 50%) at one kind of immune cell were also extracted^66–68^. Lastly, 150 highly expressed ICPs and 1493 highly expressed lncRNAs in immune cell types were extracted.

### Tumor patients collection and screen from TCGA

We screened tumor patients with high immune infiltration based on two datasets (Figure S1b). Transcriptome data and clinical information of tumor patients were accessed from databases, The Cancer Genome Atlas (TCGA, https://portal.gdc.cancer.gov) via Illumina-HiSeq platform. ESTIMATE (estimation of stromal and Immune cells in malignant tumor tissues using expression data) algorithm was used to calculate immune scores, stromal scores and estimate scores^69^. The tumor patients with top 75% immune scores were screened for follow analysis. SsGSEA was performed to validate immune infiltration of tumor patients based on previous immune gene sets.

### Identification of immune-related lncRNAs and ICPs between immunologic constant of rejection genes high and low group

TCGA tumor patients were clustered to two groups including high and low immune infiltration groups based on 20 immunologic constant of rejection (ICR) genes expression^70^ using consensus clustering algorithm based on ConsensusClusterPlus package in R. Immune-related lncRNAs and ICPs were identified between high and low ICR gene groups using t-test (Figure S1c, P < 0.05). Up-regulation represented ICPs or lncRNAs were higher expression in high-ICR group compared to low-ICR group (Fold change value > 1). Down-regulation represented ICPs or lncRNAs were lower expression in high-ICR group compared to low-ICR group (Fold change value < 1).

### Screen of significantly correlated ICPaLncCRPs in highly immune infiltration tumor samples

A large number of studies had reported that co-expressed genes tend to perform common functions. We considered that if co-expressed ICPs and ICPaLncs also could perform common or similar immunity functions. Two kinds of correlation methods including Pearson Correlation coefficients (PCCs) and mutual information (MI) analysis were used to screen significantly correlated ICP-lncRNA pairs based on above immune-related ICPs and lncRNAs (Figure S1d). The ICP-lncRNA pairs which their absolute values of PCCs were larger than 0.3 and P values were smaller than 0.05 were considered as significantly correlated ICP-lncRNA pairs. These significantly correlated ICP and lncRNA pairs were considered as ICPaLncCRPs. The ICPaLncCRPs would be distinguished positive (P < 0.05 and PCC > 0.3) and negative pairs (P < 0.05 and PCC < −0.3).

### Obtain and analysis of scRNA-seq data for ICPaLncCRPs in SKCM

ScRNA-seq expression profiles of SKCM (GSE148190^71,72^) contains 13,659 cells were downloaded from GEO. The preprocessed gene expression matrix and cell annotation information were encapsulated using the R package Seurat. Marker genes of specific cell types in SKCM and melanoma collected from published literature^71^ were used to define cell clusters. The ‘GSVA’ package is used for single sample gene set enrichment analysis (ssGSEA) to evaluate the gene set enrichment score of each cell.

### Survival analysis for important ICPaLncCRPs in multiple cancer types

A systematic survival analysis pipeline was performed for each significantly ICPaLncCRPs to verify if they were associated with prognosis. First, the cancer samples were randomly divided into two independent groups. Next, a multivariate cox regression model was used for each ICPaLncCRP to obtain a standardized cox regression coefficient for the first group. Age and sex also became confounders in this process. A risk score formula was established for each cancer patient based on the expression values of each selected gene for the held-out group weighed by their estimated regression coefficients, following the above multivariate Cox regression analysis. Thus, to avoid the overfitting, the risk scores were constructed by holding back a part of the cancer dataset during the cox regression analysis and using the held-out samples to validate the model. The 1000 permutation was performed to extract significant (P < 0.05) survival-related ICPaLncCRPs. GSE65904^73,74^ which included expression and survival of 150 SKCM samples were used to validate the survival analysis.

### Establishment and validation of the nomogram for ICPaLncCRPs

The nomogram was established based on the independent predictors of 3- and 5-years overall survival (OS) in the multivariate analysis for SKCM. The significant variables from the multivariate models were introduced to draw the graphical nomogram by utilizing “rms” and “nomogramEx” packages^75^. The calibration curves for probability of OS showed that match condition between prediction by nomogram and actual observation^76^.

### Estimation of potential for ICPaLncCRPs to predict immunotherapy response

Two SKCM datasets including TCGA and GSE35640 were used for estimating potential for ICPaLncCRPs to predict immunotherapy response (Figure S1e). The SKCM patients in GSE35640 were grouped as responders and non-responders for ICI treatment. Uniform Manifold Approximation and Projection (UMAP) was performed to distinguish responders and non-responders for ICI based on expression of ICPaLncCRPs in GSE35640. The expression of 477 prognosis-associated ICPaLncCRPs were identified in GSE35640 and calculated for a comprehensive risk score based on ssGSEA. The predicted machine learning model based on risk score was established to predict responders and non-responders in training dataset GSE35640 and testing dataset (TCGA SKCM) using support vector machine (SVM). We also extract fewer ICPaLncCRPs to predict ICI response based on Least Absolute Shrinkage and Selection Operator (LASSO) regression. Receiver operating characteristic (ROC) of SVM was used to evaluate the predicted ability based on GSE35640. Five another independent datasets including ICI response were used to validate the model^77–79^.

### Abbreviations list

lncRNAs: Long non-coding RNAs; ICP: immune checkpoints; ICI: ICP inhibitors; ICPaLncCRPs: ICP and lncRNA cooperative regulation pairs; ceRNA: competing endogenous RNA; ICR: immunologic constant of rejection; ICPaLnc: ICP-associated lncRNA; TCGA: The Cancer Genome Atlas; GEO: Gene Expression Omnibus; BLCA: Bladder Urothelial; BRCA: Breast invasive carcinoma; CHOL: Cholangiocarcinoma; COAD: Colon adenocarcinoma; ESCA: Esophageal carcinoma; GBM: Glioblastoma multiforme; HNSC: Head and Neck squamous cell carcinoma; KIRC: Kidney renal clear cell carcinoma; LIHC: Liver hepatocellular carcinoma; OV: Ovarian serous cystadenocarcinoma; PRAD: Pancreatic adenocarcinoma; READ: Rectum adenocarcinoma; STAD: Stomach adenocarcinoma; SKCM: Skin Cutaneous Melanoma; LUSC: Lung squamous cell carcinoma; LUAD: Lung adenocarcinoma; THCA: Thyroid carcinoma; UCEC: Uterine Corpus Endometrial Carcinoma; OS: overall survival; PCC: Pearson Correlation coefficients; MI: mutual information; UMAP: Uniform Manifold Approximation and Projection; SVM: support vector machine; LASSO: Least Absolute Shrinkage and Selection Operator

### Supplementary information

Supplementary Information

## Data Availability

The data that support the findings of this study are available from the TCGA (https://portal.gdc.cancer.gov/projects). Immune cell expression profiles were obtained from Gene Expression Omnibus (GEO) with the accession number GSE42058^33,34^, GSE49910^35,36^, GSE51540^37,38^, GSE59237^39,40^, GSE6863^41,42^, GSE8059^43,44^, GSE13906^45,46^, GSE23371^47,48^, GSE25320^49,50^, GSE27291^51,52^, GSE27838^53,54^, GSE28490^55,56^, GSE28698^57,58^, GSE28726^59,60^, GSE37750^61,62^ and GSE39889^63,64^. GSE148190^71,72^ was used to perform scRNA-seq expression profiles in SKCM. Independent dataset GSE65904^73,74^ was used to validate survival analysis. Immunotherapy response data was obtained from GEO with the accession number GSE35640^27,28^. The analysis results associated with this paper is available in the figshare repository^80^.

## Code availability

The R code used in the analysis of the data is available in GitHub (https://github.com/GaoYueWorkspace/ICP-related-lncRNAs/) or figshare^80^.
