# Supplementary material for: Identifying immune checkpoint-related lncRNA biomarkers for immunotherapy response and prognosis in cancers
Source: Sci Data. 2023 Sep 28;10:663. doi: 10.1038/s41597-023-02550-z (PMC10539355; doi:10.1038/s41597-023-02550-z)
Supplement: Supplementary file 1 — Supplementary Information [file 41597_2023_2550_MOESM1_ESM.pdf]

Figure S1

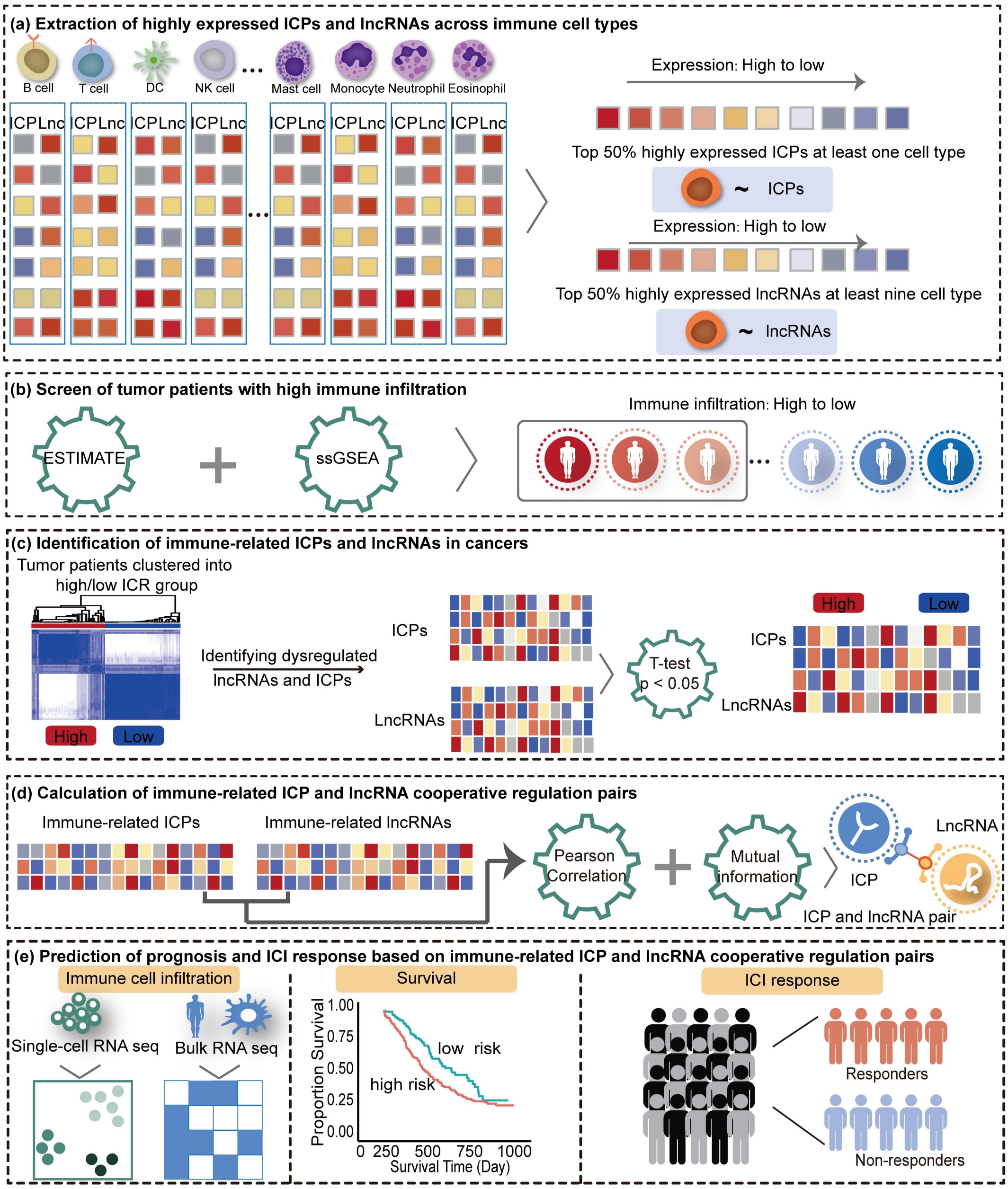

Figure S1. The system workflow of identifying and surveying ICPaLncCRPs in cancers.

Figure S2

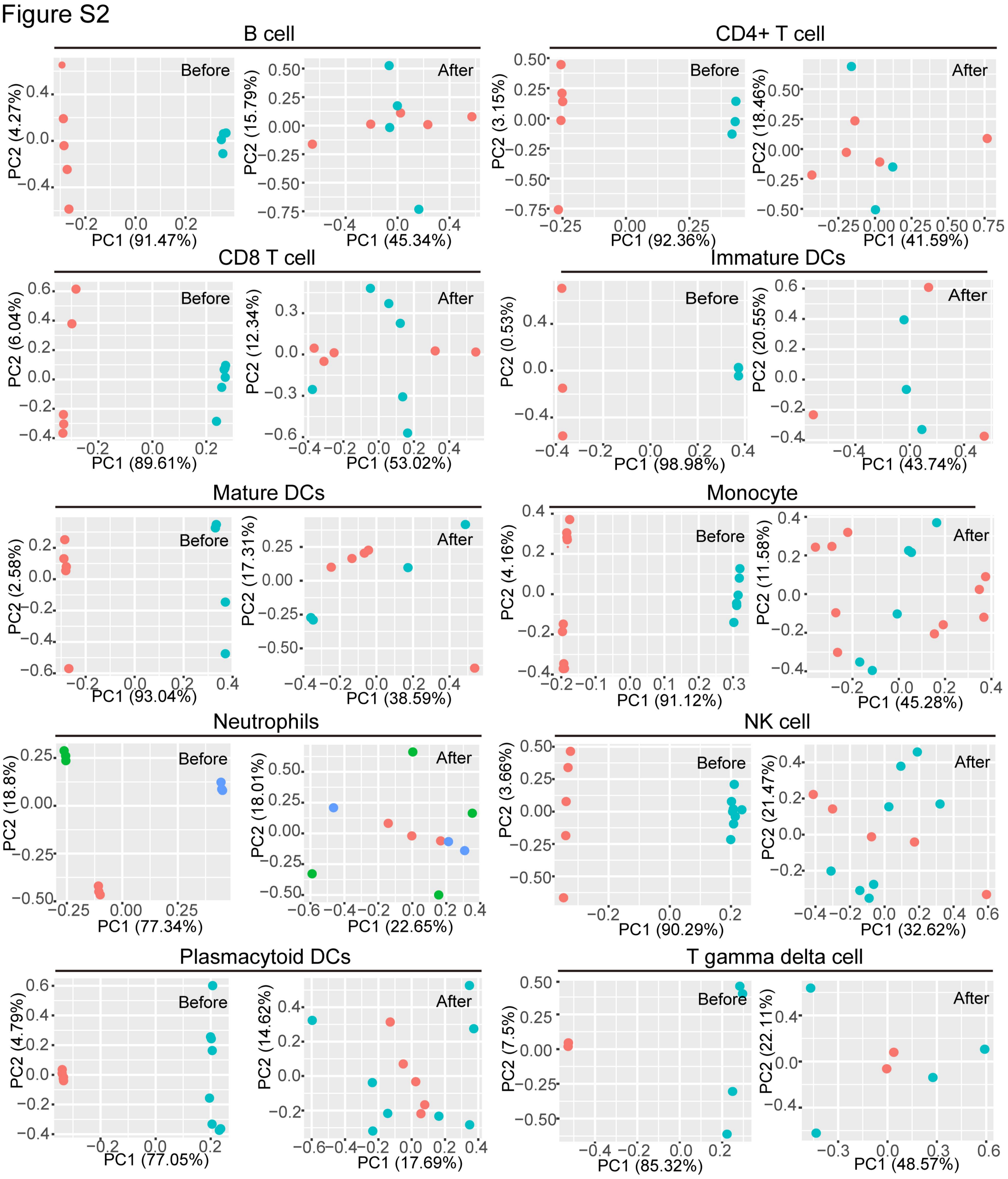

Figure S2. The sample distribution before and after removing batch effect. The point plot show two datasets (pink and blue-green) before and after removing batch effect across immune cell types.

Figure S3

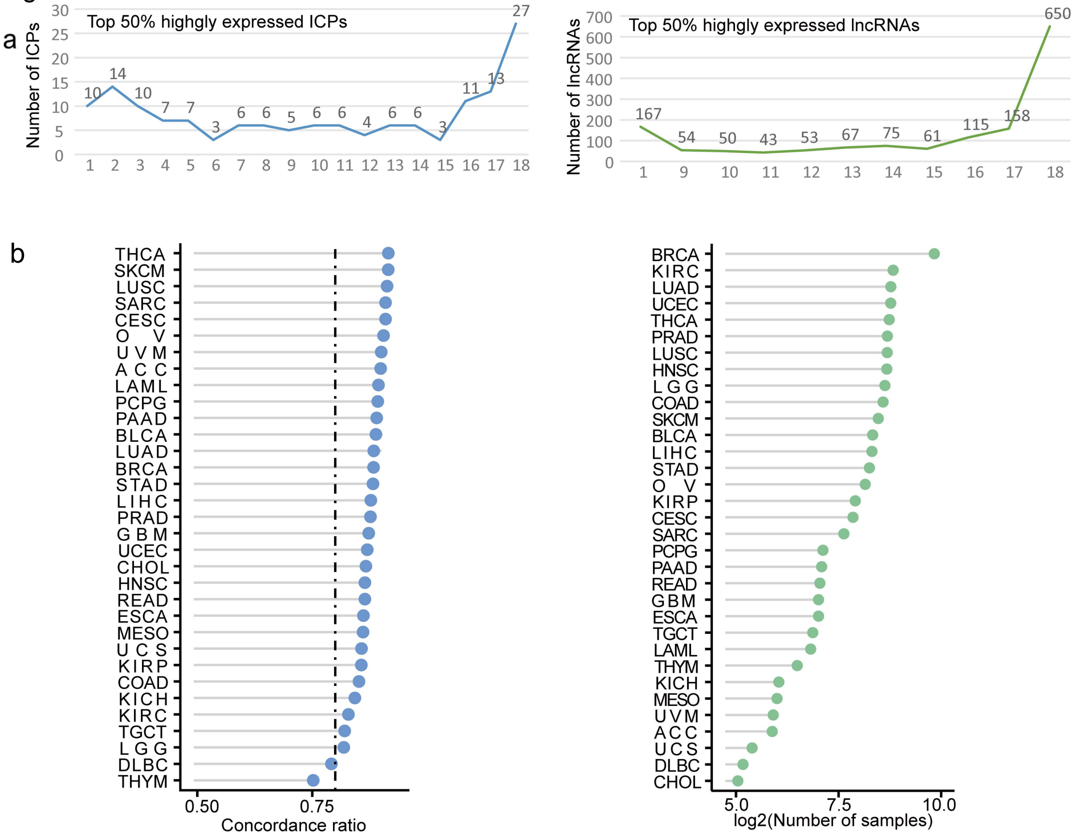

Figure S3. The highly expressed ICPs and lncRNAs across cancer types. **(a)** The line plots show numbers of highly expressed ICPs and lncRNAs in different immune cell types. **(b)** The lollipop charts show concordance ratio and intersected samples for extracting samples with high immune infiltration based on ESTIMATE and ssGSEA.

Figure S4

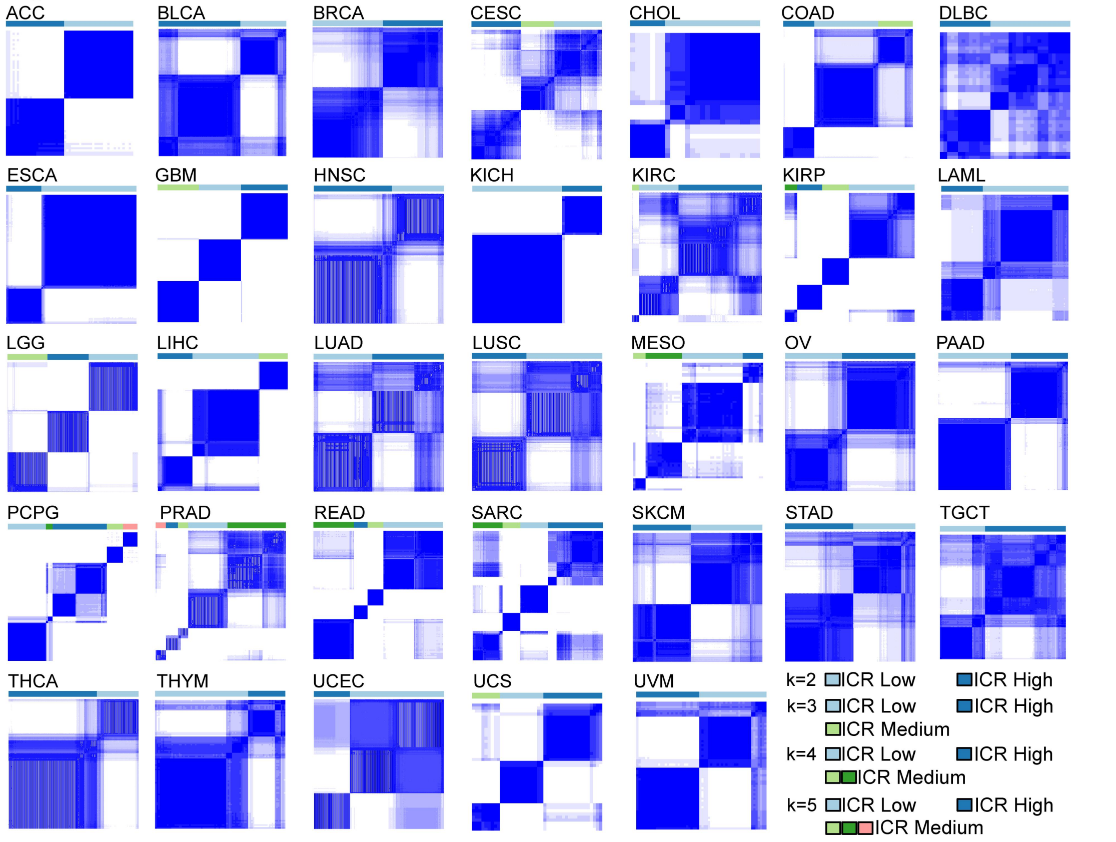

Figure S4. Consensus Clustering distinguishes TCGA samples to high and low ICR groups.

Figure S5

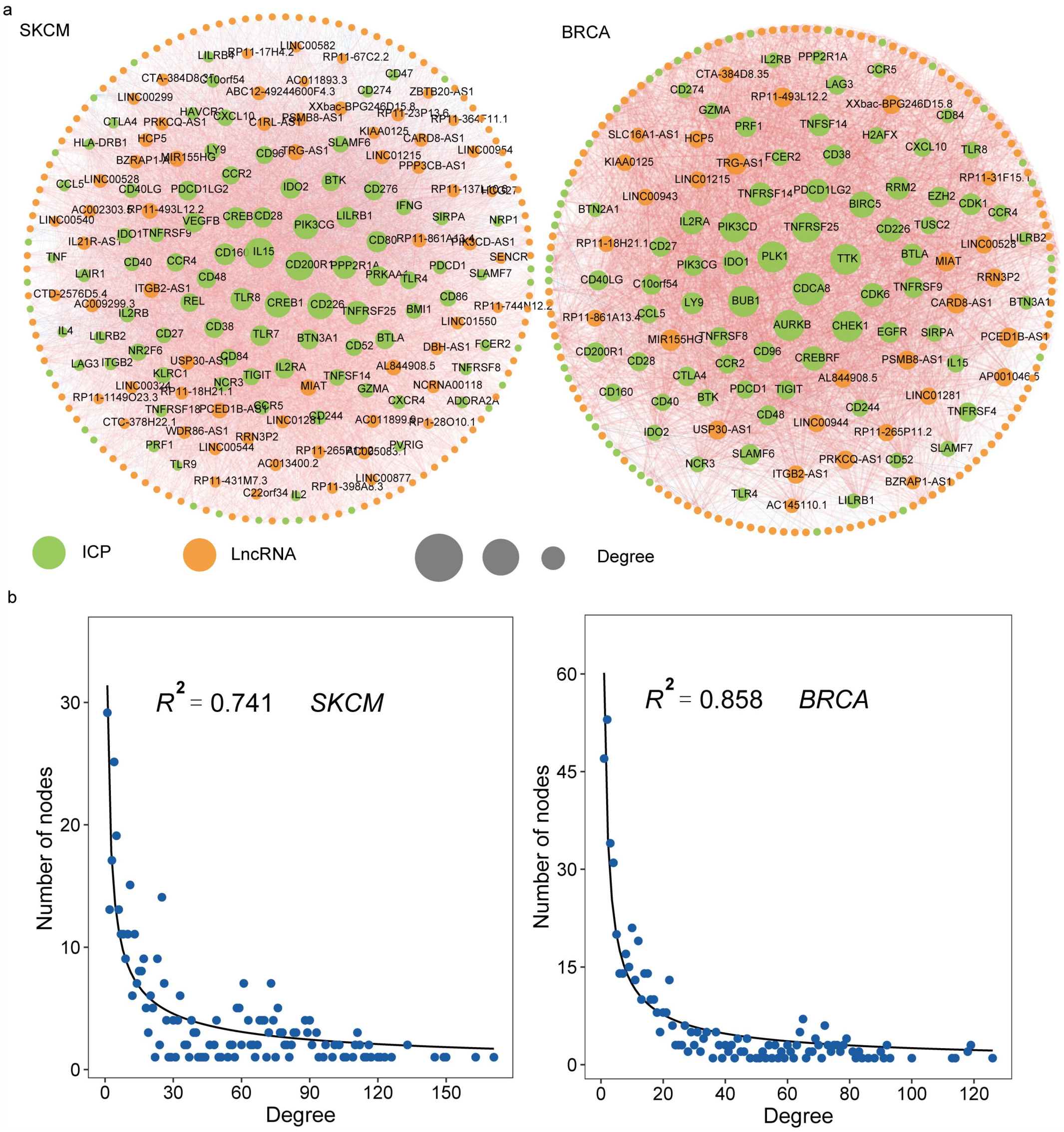

Figure S5. Immune-related ICP and lncRNA co-expressed network follow the power-law distribution. (a) Immune-related ICP and lncRNA co-expressed network in BRCA and SKCM. Green and orange nodes represent ICPs and lncRNAs. Red and blue edges represent positive and negative correlations. **(b)** X- and Y-axis of scatter chart represent degree and numbers of nodes.

Figure S6

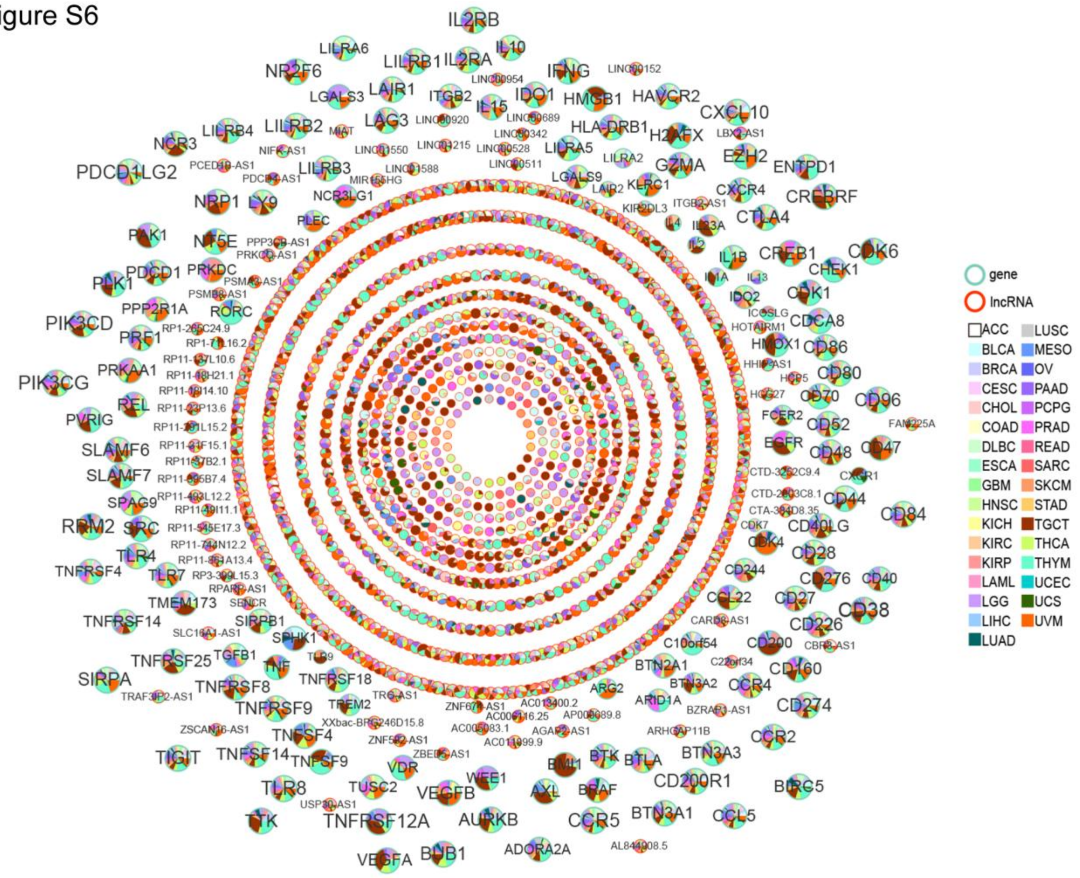

Figure S6. The hubs network in pan-cancer. Green and red nodes on the outer circle indicate ICPs and ICP-associated lncRNAs, a color pie chart shows cancers in which the ICP or ICP-associated lncRNA occur and size of the node shows the degree of nodes. An edge indicates a cooperative regulation between ICPs and ICP-associated lncRNAs.

Figure S7

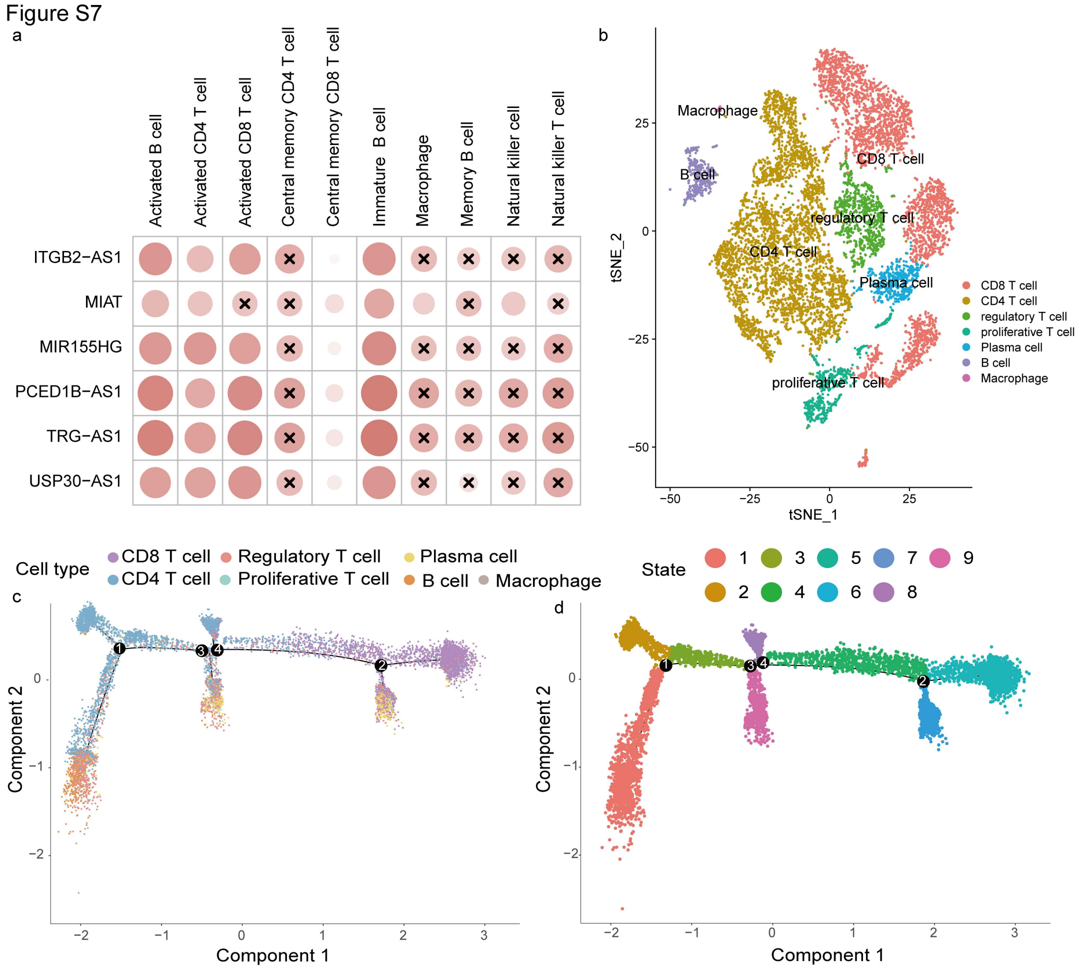

Figure S7. ICPaLncCRPs are correlated with immune cell infiltration in BRCA and SKCM. (a) The Spearman's rank correlation coefficients between hub lncRNAs and immune cell types in BRCA of which expression is significantly related to immune cell infiltration. **(b)** tSNE plot of immune cells, color-coded by diverse immune cell types for SKCM. **(c-d)** Trajectory reconstruction of all single cells based on cell types and state.

Figure S8

a

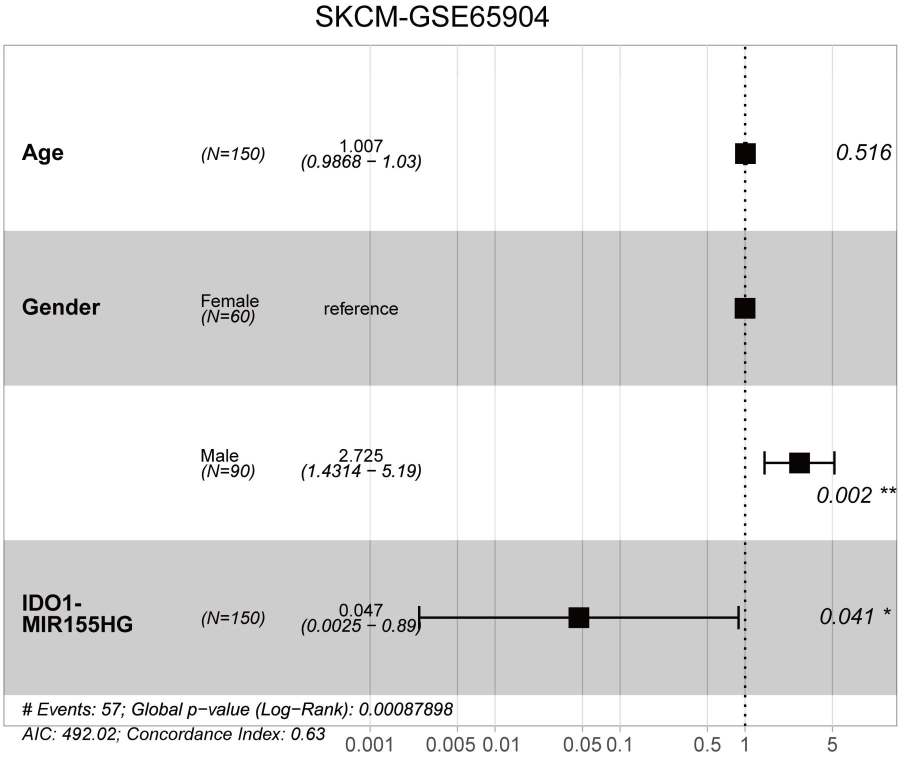

b

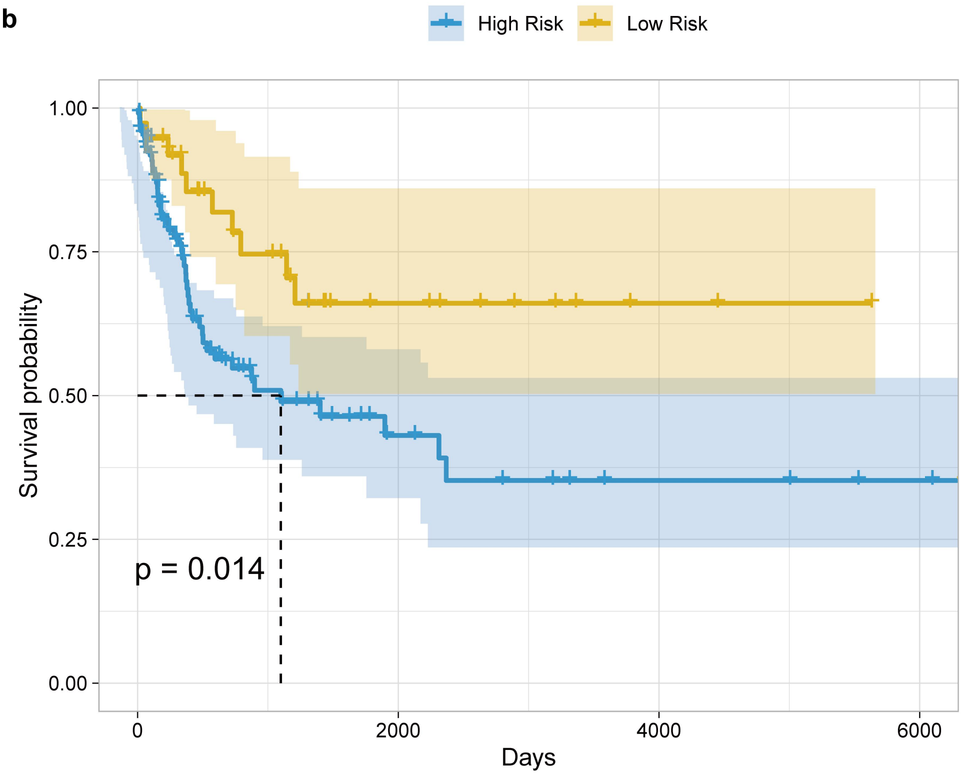

Figure S8. Survival analysis validated by another independent dataset in SKCM. (a) The forest plot for IDO1-MIR155HG. **(b)** The KM survival analysis of the OS for patients with high (blue) and low (yellow) risk scores.

Figure S9

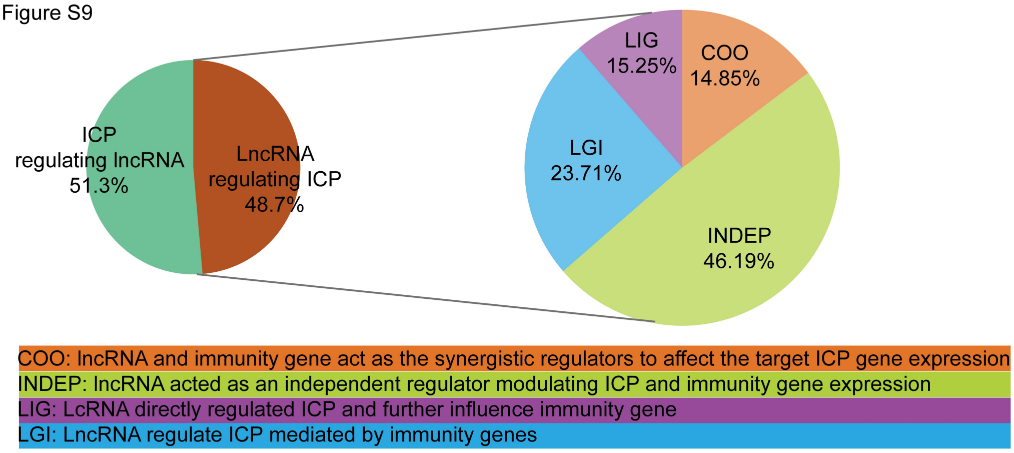

Figure S9. Possible causal inferences between lncRNAs and ICPs in ICPaLncCRPs. The pie chart in left shows percent of patterns including ICP regulating lncRNA and lncRNA regulating ICP. The pie chart in right shows four kinds of possible patterns for pattern about lncRNA regulating ICP.
